# *Helicobacter pylori*-induced immune priming requires type I interferon signaling

**DOI:** 10.3389/fimmu.2026.1942802

**Published:** 2026-10-02

**Authors:** Zhigang Rao, Christof Regl, Theresa Neuper, Hieu-Hoa Dang, Gunda Ueblagger, Andreas Völkerer, Christian G. Huber, Jutta Horejs-Hoeck

**Affiliations:** 1 Department of Biosciences and Medical Biology, Paris-Lodron University Salzburg, Salzburg, Austria; 2 Center for Tumor Biology and Immunology (CTBI), Paris-Lodron University Salzburg, Salzburg, Austria; 3 Life Sciences Center Salzburg (LSCS), Salzburg, Austria; 4 General Hospital Oberndorf, Teaching Hospital of the Paracelsus Medical University, Salzburg, Austria

**Keywords:** Helicobacter pylori, hyperresponsiveness, immune priming, monocyte, type I interferon

## Abstract

*Helicobacter pylori* (*H. pylori*) infection induces persistent colonization and chronic inflammation, yet the mechanisms by which bacterial exposure establishes sustained inflammatory responses remain incompletely understood. We previously demonstrated that priming human monocytes with viable, but not heat-killed *H. pylori*, establishes immune priming, characterized by a heightened response to subsequent LPS challenge. Here, we delineate the signaling pathways that establish this inflammatory adaptation. Unbiased whole-cell proteomics identified type I and type II interferon (IFN) signaling as the dominant pathways distinguishing monocytes exposed to viable versus heat-killed bacteria. Consistently, viable *H. pylori*, but not heat-killed or PFA-inactivated bacteria, activated IFN signaling and imprinted LPS hyper-responsiveness. Manipulating IFN signaling at the time of LPS challenge did not modify this hypersensitivity, whereas blocking type I IFN signaling during priming markedly attenuated the hyperresponsiveness. Accordingly, supplementation with type I, but not type II IFN, during priming restored hypersensitivity in monocytes exposed to PFA-inactivated *H. pylori*. Interestingly, type I IFN only partially restored LPS-hypersensitivity in monocytes exposed to heat-killed *H. pylori*, indicating that heat inactivation interferes with additional signaling cues derived from intact bacteria, which are required to drive the full hyper-responsiveness to LPS. Collectively, our findings identify type I IFN as a central regulator of *H. pylori*-induced immune priming and reveal a previously unrecognized mechanism by which *H. pylori* infection programs persistent inflammatory adaptation in host immune cells through type I IFN signaling.

## Introduction

Although initially considered a hallmark of antiviral immunity, activation of type I interferon (type I IFN) signaling has been recognized as a common feature of many bacterial infections, in which type I IFNs are induced upon sensing of pathogen-associated molecular patterns (PAMP) by pattern-recognition receptors (PRR) (1). Type I IFNs bind to and activate the canonical Janus kinase (JAK)- signal transducers and activators of transcription (STAT) signaling, resulting in phosphorylation of STAT1 and STAT2 and formation of the interferon-stimulated gene factor 3 (ISGF3) complex with IRF9, thereby driving the transcription of interferon-stimulated genes (ISGs) (2). Given the ubiquitous expression of interferon-α/β receptor (IFNAR), type I IFN signaling exerts broad immunomodulatory effects (3), while type II interferon (type II IFN) signaling likewise serves as a major regulator of antimicrobial immunity and inflammatory responses (4). Despite signaling through distinct receptors and activating different STAT complexes, type I IFN and type II IFN signaling share substantial overlap in downstream gene expression and frequently cooperate to shape innate immune functions (5).

While certain intracellular pathogens exploit type I IFN signaling to promote their survival (6–8), accumulating evidence identifies its prominent roles in immunomodulatory (9) and immune priming and memory (10). For instance, type I IFN-dependent reprogramming has been implicated in the regulation of hematopoietic stem and progenitor cell responses during *Mycobacterium tuberculosis* infection (11), in β-glucan-induced hematopoietic expansion (12), and in shaping the magnitude of peripheral blood mononuclear cells (PBMC) or macrophage responses to secondary inflammatory stimulation (13–16). These observations indicate that type I IFN signaling can induce long-lasting functional adaption of innate immune cells beyond its established role in antimicrobial defense. Therefore, it is plausible to speculate that type I IFN contributes to inflammatory adaptation during chronic bacterial infections, such as *Helicobacter pylori* (*H. pylori*), the infection of which usually establishes life-long colonization of the gastric mucosa and drives persistent inflammation that contributes to chronic disease progression (17).

Accordingly, our previous work demonstrated that *H. pylori* priming induces immune priming in human monocytes, characterized by exaggerated TNF-α production following subsequent lipopolysaccharide (LPS) challenge (18, 19). Unlike many gram-negative pathogens, *H. pylori* LPS is structurally modified and exhibits low or absent toll-like receptor (TLR)4 agonist activity, allowing the bacterium to evade robust TLR4-driven inflammation (20). Instead, *H. pylori* engages alternative sensing pathways, including TLR2 during bacteria uptake (21) and TLR8 for bacterial RNA (22). Indeed, *H. pylori* has been shown to activate type I IFN responses in monocytes through TLR8 (22), highlighting interferon signaling as an integral component of the host response to *H. pylori* infection. Although *H. pylori*-induced type I IFN responses and immune priming have each been described independently, whether these two phenomena are mechanistically linked remains unknown.

In the present study, we show that type I IFN, but not type II IFN signaling during the priming phase with *H. pylori* is required for establishing enhanced responsiveness of monocytes to subsequent LPS challenge. These findings identify type I IFN signaling as a previously unrecognized regulator of *H. pylori*-induced immune priming and provide mechanistic insights into how persistent *H. pylori* infection may promote chronic inflammatory responses.

## Materials and methods

### Isolation of PBMC and monocytes

Human PBMC were isolated from fresh buffy coats prepared from freshly collected peripheral blood of healthy anonymized volunteers, provided by the Blood Bank Salzburg. Briefly, PBMC were isolated via density gradient centrifugation (836 × g, 20 min, RT) using Histopaque-1077 (Sigma-Aldrich, Vienna, Austria, #10771). Monocytes were subsequently purified using CD14 MicroBeads (Miltenyi Biotec, Bergisch-Gladbach, Germany, #130-050-201) following the manufacturer´s instructions, with the exception that only 30% of the recommended amount of MicroBeads were used. The purity of CD14^+^ cells was > 98% and the viability was > 99%, as determined by flow cytometry. Cells were cultured in monocyte medium: RPMI-1640 medium (Sigma-Aldrich, #R0083) supplemented with 10% heat-inactivated fetal calf serum (Biowest, Nuaillé, France, #S1400) and 1% L-glutamine (Sigma-Aldrich, #G7513). Following isolation, monocytes were allowed to adhere for 1–2 h at 37 °C in a humidified atmosphere containing 5% CO_2_ before further experimental use.

### Bacterial culture and infection

*H. pylori P12* wild type were cultivated under microaerophilic conditions using an Oxoid 3.5 L bacterial culture bucket (Thermo Fisher Scientific, Vienna, Austria, #AB0035A) containing Oxoid CampyGen 3.5 L sachets (Thermo Fisher Scientific, #CN0035A). Bacteria were grown on GC agar-plates supplemented with 10% horse serum (Biowest) at 37 °C. Frozen bacteria stocks were first cultured for 72 h, followed by subculturing onto fresh agar plates for another 24 h prior to use in monocyte infection experiments. Bacterial biomass was harvested from agar plates using sterile cotton buds (Paul Boettger, Bodenmais, Germany, #09129-0100) and suspended in 1 mL PBS (Sigma-Aldrich, #D8537). Optical density was determined at OD600 using a BioPhotometer Plus device (Eppendorf, Vienna, Austria, #6132) and bacteria concentrations were estimated based on an in-house OD600-to-cell number calibration curve. Unless otherwise stated, infection experiments were performed at a multiplicity of infection of 5.

Heat-killed *H. pylori* (*Hp^heat^*) was generated by incubating bacterial suspensions in PBS at 95 °C for 5 min, followed by equilibration at RT for 3 min before use. Paraformaldehyde (PFA)-inactivated *H. pylori* (*Hp^PFA^*) was prepared by treating viable *H. pylori* suspensions with 4% paraformaldehyde (Thermo Fisher Scientific, #I28800) at RT for 15 min. Subsequently, bacteria were pelleted by centrifugation (10, 000 × g, RT, 5 min), washed twice with PBS, and resuspended in PBS before further use.

For the generation of antibiotic-treated *H. pylori* (*Hp^ab^*), *H. pylori* was cultured in BHI medium (37.75 g/L) containing β-cyclodextrin (10 g/L; Thermo Fisher Scientific, #J63161), thrimetoprim (2.5 mg/L; pre-dissolved in DMF; Thermo Fisher Scientific, #T7883), vancomycin (10 mg/L; pre-dissolved in water; Roth, Karlsruhe, Germany, #0242.3), nystatin (1 mg/L; pre-dissolved in DMSO; Roth, #2411) and vitamin mix consisting of dextrose (1 g/L, #D-9434), L-glutamine (0.1 g/L, #G-3126), carboxylase (1 mg/L, #C8754), iron(III) nitrate nonahydrate (0.2 mg/L, #216828), thiamin hydrochloride (0.03 mg/L, #T-4625), 4-aminobenzoic acid sodium salt (0.13 mg/L, #A6928), β-nicotinamide adenine dinucleotide hydrate (2.5 mg/L, #N-7004), vitamin B12 (0.1 mg/L, #V-2876), L-cystine (11 mg/L, #C-6727), Adenine (10 mg/L, #A9795), Guanine (0.3 mg/L, #51030), Arginin (1.5 mg/L, A-6969), Uracil (5 mg/L, #U1128) (all from Sigma-Aldrich) and cysteine-HCl (0.26 g/L, Roth, #1694.2). Viable *H. pylori* (OD600 = 0.5 – 1) cultures were exposed to amoxicillin (0.05 mg/mL; pre-dissolved in DMSO; MedChemExpress, Monmouth Junction, NJ, #HY-B0467A) and clarithromycin (0.05 mg/mL; pre-dissolved in DMSO; MedChemExpress, #HY-17508) for 2 h. Following treatment, bacteria were collected by centrifugation (10, 000 × g, 5 min, RT), washed twice with PBS, and resuspended in PBS before use.

To verify the loss of cultivability, *Hp^heat^*, *Hp^PFA^* and *Hp^ab^* preparations were replated onto GC agar-plates supplemented with 10% horse serum and incubated under microaerophilic conditions at 37 °C. No colony formation was detected within 72 h of incubation.

### Monocyte short-term immune priming model

The human monocyte short-term immune priming model was established as previously described (18, 19). In brief, monocytes were seeded at a density of 1 × 10^6^/mL (100 µL/well) in 96-well V-bottom cell culture plates (Greiner Bio-One, Kremsmünster, Austria, #651180) and rested for 1 h. Afterwards, cells were either left unprimed, or exposed to indicated *H. pylori* conditions at a multiplicity of infection of 5 for 24 h. Following the priming phase, plates were centrifugated (716 × g, RT, 5 min), and supernatants (200 µL) were collected for measurement of cytokines. Cells were then gently washed with 100 µL of challenge medium: RPMI-1640 medium (Sigma-Aldrich, #R0083) supplemented with 10% heat-inactivated fetal calf serum (Biowest, #S1400) and 1% L-glutamine (Sigma-Aldrich, #G7513) and 1% penicilin/streptomycin (Sigma-Aldrich, #P4333). Thereafter, cells were incubated in 200 µL challenge medium in the absence or presence of 10 ng/mL *E. coli* LPS (O55:B5, Sigma-Aldrich, #L2880) for another 24 h. At the end of the experiment, supernatants were collected for measurement of cytokines.

To access the viability of *H. pylori* after priming and challenge, supernatants were collected 1) after priming and 2) after the subsequent challenging phase, and plated on GC agar plates supplemented with 10% horse serum under microaerophilic conditions at 37 °C for 6 days. Colony formation was monitored by photography.

For pharmacological and cytokine intervention experiments, celecoxib (1 – 10 µM; MedChemExpress, #HY-14398), PGE_2_ (1–10 nM; MedChemExpress, #HY-101952), IFN-α (10 ng/mL; Immuno Tools, Friesoythe, Germany, #11343504), IFN-β (10 ng/mL; Immuno Tools, #11343594), anifrolumab (20 ng/mL; MedChemExpress, #HY-P99168), IFN-γ (20 ng/mL; Immuno Tools, #11343534) were added either 15 min before the onset of the priming phase or shortly before the challenge phase, as indicated in the respective experiment.

### Determination of cytokine levels

Cell culture supernatants were collected following monocyte stimulation and stored at -80 °C before analysis. Concentrations of tumor necrosis factor (TNF)-α and interferon gamma-induced protein 10 (IP-10) were quantified using commercially available Enzyme-linked Immunosorbent Assay kits (TNF-α: #900-K25K; IP-10: #900-K39; Peprotech, London, United Kingdom) according to the manufacturer´s instructions.

### Whole-cell proteomic analysis and bioinformatics

Monocytes were infected with the corresponding *Hp* conditions for 24 h. Subsequently, cell pellets (1.5 × 10^6^) were harvested and processed as previously described (21). Briefly, pellets were resuspended in 50 mM triethylammonium bicarbonate buffer (pH 7.55, Sigma-Aldrich, #T7408) containing 5% SDS (Sigma-Aldrich, #L3771) and 1 × cOmplete Mini EDTA-free protease inhibitor cocktail (Roche, #04693159001). Samples were then heated at 95 °C for 5 min, followed by sonication for 5 min using a Bioruptor device (Diagenode, Liège, Belgium). After centrifugation (14, 000 × g, 4 °C, 1 min), supernatants were harvested and protein concentrations were determined using the Pierce BCA Protein assay kit (Thermo Fisher Scientific, #23255) according to the manufacturer’s instructions. Protein lysates were then treated with tris-(2-carboxyethyl)-phosphine-hydrochloride (5 mM, Sigma-Aldrich, #75259) at 55 °C for 15 min to reduce disulfides, followed by alkylation of the cysteine residues with iodoacetamide (Sigma-Aldrich, #I6125) at a final concentration of 20 mM for 10 min at 22 °C in the dark. Samples were then acidified to pH ≤ 1 using 12% (v/v) orthophosphoric acid (Merck, Darmstadt, Germany, #100573), and proteins were precipitated by addition of 100 mM triethylammonium bicarbonate buffer (pH 7.5, in 90% methanol) (Merck, #106009) at a ratio of 7:1 (v/v). Protein purification was then performed by suspension trapping using S-Trap mini columns (Protifi, Huntington, NY, USA) according to the manufacturer’s instructions. Proteins were digested to peptides using sequencing grade modified porcine trypsin (Promega, Madison, WI, USA, #V5111) at a protease/protein ratio of 1:10 (w/w) at 37 °C for 16 h. Resulting peptides were dried in a vacuum centrifuge at 60 °C and resuspended in an aqueous solution containing 1% acetonitrile (VWR International, Vienna, Austria, #20060.320) and 0.1% formic acid (Sigma-Aldrich, #1002631000) (v/v) to a final concentration of 3.0 µg/µL.

Chromatographic separation of 6.0 µg of peptides was performed by a reversed-phase nanoHPLC using a Vanquish Neo UHPLC System (Thermo Fisher Scientific), equipped with an Aurora Ultimate XT C18 column (250 × 0.075 mm i.d.) (ionopticks, #AUR3-25075C18-XT). Separation was conducted at a flow rate of 300 nL/min using 0.1% aqueous formic acid (solvent A) and 0.1% formic acid in acetonitrile (solvent B) with the following gradient: 1% B for 5 min, followed by a linear gradient of 1-35% B till 100 min. This was followed by a washing step with 80% B for 5 min and column re-equilibration at 1% B for 15 min. Column temperature was maintained at 50 °C, while the autosampler was kept at 7 °C.

The nanoHPLC system was coupled to a Q Exactive™ Hybrid Quadrupole-Orbitrap™ mass spectrometer (Thermo Fisher Scientific) via a Nanospray Flex™ ion source (Thermo Fisher Scientific). Spray voltage was set to 1.5 kV, the S-lens RF level to 60.0 and the capillary temperature to 250 °C. Each acquisition cycle consisted of one full MS scan over a *m/z* range of 350–1, 200 at a resolution of 35, 000 at *m/z* 200, followed by 36 data-independent higher-energy collisional dissociation scans in 24.0 *m/z* isolation windows from *m/z* 350–1200 with 29% normalized collision energy and a resolution of 17, 500 at *m/z* 200. For all scans, the automatic gain control target was set to 1e6 charges with a maximum injection time of 60 ms. Data acquisition was performed using Thermo Scientific™ Chromeleon™ 7.2 CDS (Thermo Fisher Scientific).

For data analysis, DIA-NN 2.2.0 (23) was used with the protease settings “Trypsin/P” with a maximum of three missed cleavages, carbamidomethylation of cysteine as fixed and oxidation of methionine as variable modification. Protein identification was performed against an in-silico generated spectral library of the reviewed Swiss-Prot UniProt consortium database (24) containing Homo sapiens (Human) entries downloaded on 22.07.2025, with a false discovery rate of 1%. The resulting protein groups were further processed in RStudio 2525.05.1 + 513.pro3 (25). First, the intensities were log2-transformed, followed by the removal of proteins that were not detected across at least 10% of all samples, and potential contaminants. Next, intensities were normalized by subtraction of the median intensity of each sample, followed by a principal component analysis. As principal component analysis revealed clustering associated not only with the treatment but also with donor availability, differential expression analysis was performed with *limma* in R using the donor as a blocking factor for paired analysis (26). The *p*-values were adjusted with the Benjamini-Hochberg procedure. Fast gene set enrichment analysis was performed using *fgsea* package (27) in R using the Reactome pathway database (https://reactome.org/cite).

The mass spectrometry proteomics data have been deposited to the ProteomeXchange Consortium (http://proteomecentral.proteomexchange.org) via the PRIDE partner repository (28) with the dataset identifier PXD080861.

### Sample preparation and analysis of PGE_2_

Monocytes (1 × 10^6^ in 0.5 mL culture medium) were pre-treated without or with 10 µM celecoxib for 15 min, and then infected without or with viable *H. pylori*, or *Hp^heat^* for 24 h. Cell supernatants (0.4 mL) were harvested in 0.5 mL of ice-cold methanol containing 800 pg of d_4_-PGE_2_ (Fisher Scientific, 30427024) as an internal standard to facilitate metabolite quantitation and sample recovery. Solid phase extraction was conducted using adapted protocols as previously described (29). Briefly, samples were first kept at -20 °C for at least 2 h to allow protein sedimentation, followed by centrifugation (400 × g, 4 °C, 10 min). Supernatants were transferred to new tubes and mixed with 2 mL of acidified water (pH = 3.5) before loading onto C18 AR column cartridges (15 mg, Agilent Technology) pre-equilibrated with 1 mL methanol and 0.5 mL water. After sequential washing steps with 1 mL water and 1 mL hexane, metabolites were eluted with 1 mL methanol and brought to dryness using an Eppendorf concentrator (Thermo Fisher Scientific) using the program V-HV at 30 °C for 60–90 min. Dried lipid films were stored at -20 °C before dissolving in 100 µL methanol/water (50/50, v/v) for high-performance liquid chromatography–tandem mass spectrometry (HPLC-MS/MS) analysis.

The KNAUER AZURA^®^ Analytical (U)HPLC 862 bar system with DAD detector and a quaternary pump (KNAUER, Berlin, Germany) was coupled to a Thermo Scientific Q Exactive mass spectrometer (Thermo Fisher Scientific), which was equipped with a Thermo Scientific™ Ion Exactive™ ion source with a heated Electrospray Ionization (HESI-II) source (Thermo Fisher Scientific). Metabolites were separated on a reversed phase YMC-Triart C18 column (150 × 2.0 mm i.d., 1.9 µm d_p_, 12 nm pore size; YMC group, Kyoto, Japan) at a flow rate is 0.3 mL/min at 50 °C. The mobile phase was composed of A (0.01% acetic acid in water) and B (0.01% acetic acid in methanol) (30), which started with 20% B (0-0.1 min), followed by a linear gradient of 20-95% B (0.1–3 min), then 95% B (3–5 min) and finally 20% B (5-5.1 min). This was followed by a washing step with 99% B for 5 min. The DAD detection was carried out at 195 nm.

Mass spectrometry was operated in negative ionization mode using parallel reaction monitoring. The source heater temperature was set to 300 °C, spray voltage to -3 kV, sheath gas flow to 22 arbitrary units, auxiliary gas flow of 2 arbitrary units, capillary temperature to 320 °C and S-lens RF level to 50. Normalized collision energy was set to -20 eV. The m/z values for targeted MS/MS analysis were 355.24280 (d_4_-PGE_2_) and 193.15360 (d_4_-PGE_2_-specific fragment) for d_4_-PGE_2_, and 351.21770 (PGE_2_) and 189.12850 (PGE_2_-specific fragment) for PGE_2_, with an isolation window of 0.8 m/z. The resolution was set to 17, 500 at m/z 200, the automatic gain control target was set to 1e6 charges with a maximum injection time of 150 ms. Data were acquired using Thermo Orbitrap™ Scientific™ 7.2 CDS and data analysis was performed with Skyline software (31) with a mass tolerance of 5 ppm, where the retention time was defined with the help of external lipid standards (PGE_2_ and d_4_-PGE_2_). The absolute amount of PGE_2_ was calculated from integrated signal intensities that were normalized using the deuterated internal standards and corrected by external calibration curve of PGE_2_ (linear regression based on 8 dilutions).

### RNA extraction, cDNA generation and qPCR

Total RNA from cell pellets was isolated using TRI reagent (Sigma-Aldrich, #T9424) according to the manufacturer’s instructions. Reverse transcription of extracted RNA into cDNA was conducted using RevertAid H Minus M-MuLV reverse transcriptase (Thermo Fisher Scientific, #EP0452). Quantitative real-time PCR was carried out with diluted cDNA (1 µL + 4 µL nuclease free water), Luna Universal qPCR Master Mix (5 µL; New England BioLabs, #M3003L) and forward and reverse primers (0.3 µM, each; Sigma-Aldrich; Eurofins Genomics, Ebersberg, Germany; or Biomers.net GmbH, Ulm, Germany) on a Rotor Gene 3000 device (Corbett Research). The thermal cycle started at 95 °C for 3 min, followed by 40 cycles of 95 °C for 10 s, 65 °C for 15 s, and 72 °C for 30 s. Relative mRNA expression was normalized to the expression of housekeeping gene RPLP0 and calculated as 2^−ΔCt^, with ΔCt representing the difference between cycle threshold (Ct) of the gene of interest minus the Ct of the housekeeping gene. Specificity of the primers was continuously monitored by melting curve analysis using a temperature ramp from 70 °C to 95 °C with 0.5 °C increments and 5 s hold at each step (except for 45 s after the first step at 70 °C). Primers were listed in **Table 1**.

**Table 1:** List of primers.

Primer	Forward	Reverse
IDO1	5’-gtctggctggaaaggcaacccc-3’	5’-gaactgagcagcatgtcctccacc-3’
IFNA2	5’-gtgctcagctgcaagtcaag-3’	5’-tctgctggatcatctcatgg-3’
IFNB1	5’-cttggattcctacaaagaagcagc-3’	5’-tcctccttctggaactgctgca-3’
IFNG	5’-ttggcttaattctctcggaaacga-3’	5’-gagttccattatccgctacatctga-3’
ISG15	5’-catctttgccagtacaggag-3’	5’-tgatctgcgccttcagctct-3’
IRF3	5’-tatgccctctggttctgtgtg-3’	5’-cttgtactggtcggaggtgag-3’
IRF7	5’-cgtacctgtcaccctccccaagc-3’	5’-ccgcagcagttcctccgtgtagc-3’
PTGS2	5’-gctgctgaatttaacaccctctatcactggc-3’	5’-tgcctggtgaatgattcaacaaactgggta-3’
RPLP0	5’-ggcaccattgaaatcctgagtgatgtg-3’	5’-ttgcggacaccctccaggaag-3’
SOCS1	5’-ttggagggagcggatgggtgtag-3’	5’-agaggtaggaggtgcgagttcaggtc-3’
SOCS2	5’-cggggattgagttgcacctgtatagcatgatattc-3’	5’-ccaaatcaaccaaaaaaagtgaccatgaagtcctg-3’
SOCS3	5’-tgagtatgtggctttcctatgctgggtccctct-3’,	5’-atactataccttcctgtacctgggtggatggagcg-3’;
STAT1	5’-cgagaccaatggtgtggcaaaga-3’	5’-ctgctgtctccgcttccactcaa-3’

### SDS-PAGE and western blot

Cell pellets were harvested and lysed in Pierce™ RIPA Buffer (Thermo Fisher Scientific, #89900) supplemented with 0.1 mM PMSF (Sigma-Aldrich, #93482) and cOmplete Mini EDTA-free protease inhibitor cocktail (Roche, Vienna, Austria, #04693159001), according to the manufacturer’s instructions. Protein concentrations were determined by Pierce Bradford Protein Assay Kit (Thermo Fisher Scientific, #23200). Lysates were mixed with 4 × Laemmli sample buffer (Biorad, #1610747) supplemented with 2-mercaptoethanol (10%, v/v; Sigma-Aldrich), followed by heating at 95 °C for 5 min.

Protein lysates (equal to 8-10 µg of protein) were separated on mPAGE™ 4-12% Bis-Tris Precast Gels (MERCK, Darmstadt, Germany, #MP41G15) and transferred onto nitrocellulose membranes (0.45 µm pore size, Bio-Rad, Vienna, Austria, #1620115) using a Trans-Blot transfer cell (Bio-Rad). Membranes were then blocked with 5% (v/v) non-fat milk (Roth, #T145.1) in TBS-T buffer containing 50 mM Tris-HCl (pH 7.6, Roth, #AE15.2), 150 mM NaCl (Roth, #3957.1) and 0.1% (v/v) Tween-20 (Sigma-Aldrich, #P1379) at RT for 1 h. Afterwards, membranes were incubated with primary antibodies diluted in 5% bovine serum albumin (Roth, #1003336101) (w/v) in TBS-T buffer at 4 °C on a shaker overnight, followed by incubation with horseradish-peroxidase-labeled anti-rabbit IgG (Cell Signaling, MA, USA, #7074) secondary antibody at RT for 1 h. The following primary antibodies (all from Cell Signaling) were used: rabbit monoclonal anti-β-actin (13E5), 1:10000 (#4970); rabbit monoclonal anti-IRF-9 (D8G7H), 1:1000 (#28492); rabbit anti-STAT1, 1:5000 (#9172); rabbit monoclonal anti-p-STAT1 (Tyr701), 1:5000 (#5375); rabbit monoclonal anti-STAT2 (D9J7L), 1:1000 (#72604); rabbit monoclonal anti-p-STAT2 (Y690), 1:1000 (#88410); rabbit monoclonal anti-STAT3 (79D7), 1:1000 (#4904); rabbit monoclonal anti-p-STAT3 (Tyr705, D3A7), 1:1000 (#9145).

For detection, membranes were incubated in West Pico PLUS chemiluminescent substrate (Thermo Fisher Scientific, #34579) at RT for 2 min and detected by a ChemiDoc imaging device (Bio-Rad). Data from densitometric analysis were background-corrected and quantified using Fiji (ImageJ 1.54p).

### Statistical analysis

Data are presented as mean, mean +/± SD, or as single data of *n* independent experiments from individual donors, sample size is indicated in the figure legends. Outliers were detected using Grubbs’ test (significance level of alpha = 0.05). Z-scores were calculated by standardizing each value to the mean and standard deviation of the corresponding dataset. Statistical analyses were performed using GraphPad Prism 11 Software (GraphPad Software, San Diego, CA). Two-tailed paired *t*-tests were used for comparison of two groups. Differences between multiple treatment groups were analyzed using repeated measures one-way or two-way ANOVA, or mixed effects model, followed by Tukey’s or Šídák’s *post-hoc* tests, as indicated in the figure legends. The criterion for statistical significance is *p* ≤ 0.05 (*p ≤ 0.05, **p ≤ 0.01, ***p ≤ 0.001, ****p ≤ 0.0001).

## Results

### Activation of interferon signaling is a hallmark of infection with viable *H. pylori*

Our previous work demonstrated that active *H. pylori* infection is required to potentiate subsequent immune responses (18), as evidenced by the inability of heat-killed *H. pylori* (*Hp^heat^*) to enhance monocyte responsiveness to subsequent LPS, whereas viable *H. pylori* was sufficient to significantly increase TNF-α secretion upon secondary LPS-challenge (**Figure 1a**). Notably, unchallenged cells did not release TNF-α (**Figure 1a**), suggesting that any residual bacteria or bacterial factors present after the priming phase are not sufficient to induce cytokine production on their own. This is supported by the fact that *H. pylori* colonies were observed only on agar plates incubated with the supernatant after priming (**Supplementary Figure S1a**), but not after the challenge phase (**Supplementary Figure S1b**). To gain mechanistic insight into this phenomenon, we performed whole-cell proteomic analysis of human primary monocytes following 24 h stimulation with either viable *H. pylori* or *Hp^heat^*. Principal component analysis revealed distinct clustering between the two conditions, indicating substantial differences in the global proteomic profiles (**Figure 1b**). These differences are further illustrated by a volcano plot highlighting differentially regulated proteins between the two treatment groups (**Figure 1c**). Next, we performed Gene Set Enrichment Analysis (32) to identify significantly differentially regulated pathways between the two conditions. Compared to viable *H. pylori*, *Hp^heat^* induced upregulation of pathways related to slc mediated transmembrane transport and insulin like growth factor transport and uptake, whereas IFN signaling pathways were downregulated (**Figure 1d**). However, proteins associated with the upregulated pathways showed only modest differences between *Hp^heat^* and viable *H. pylori* (**Supplementary Figures S2a, b**), with elevated complement component 3 (C3) expression and a trend of increase for apolipoprotein A1 (APOA1) in *Hp^heat^*-treated monocytes (**Supplementary Figure S2c**). Notably, viable *H. pylori* induced the expression of a large set of proteins involved in IFN signaling, including MX dynamin like GTPase 2 (MX2), indoleamine 2, 3-dioxygenase (IDO1), 2’-5’-oligoadenylate synthetase 2 (OAS2), STAT1 and various human leukocyte antigen (HLA) proteins, whereas their levels remained largely unchanged in *Hp^heat^*-treated conditions compared to untreated controls (Ctrl) (**Figures 1e, f**). In fact, *H. pylori* has been reported to activate type I IFN signaling across multiple cell types, including primary epithelial cells (33) and immune cells such as human monocytes (18, 22) as well as dendritic cells (21, 34).

**Figure 1:**
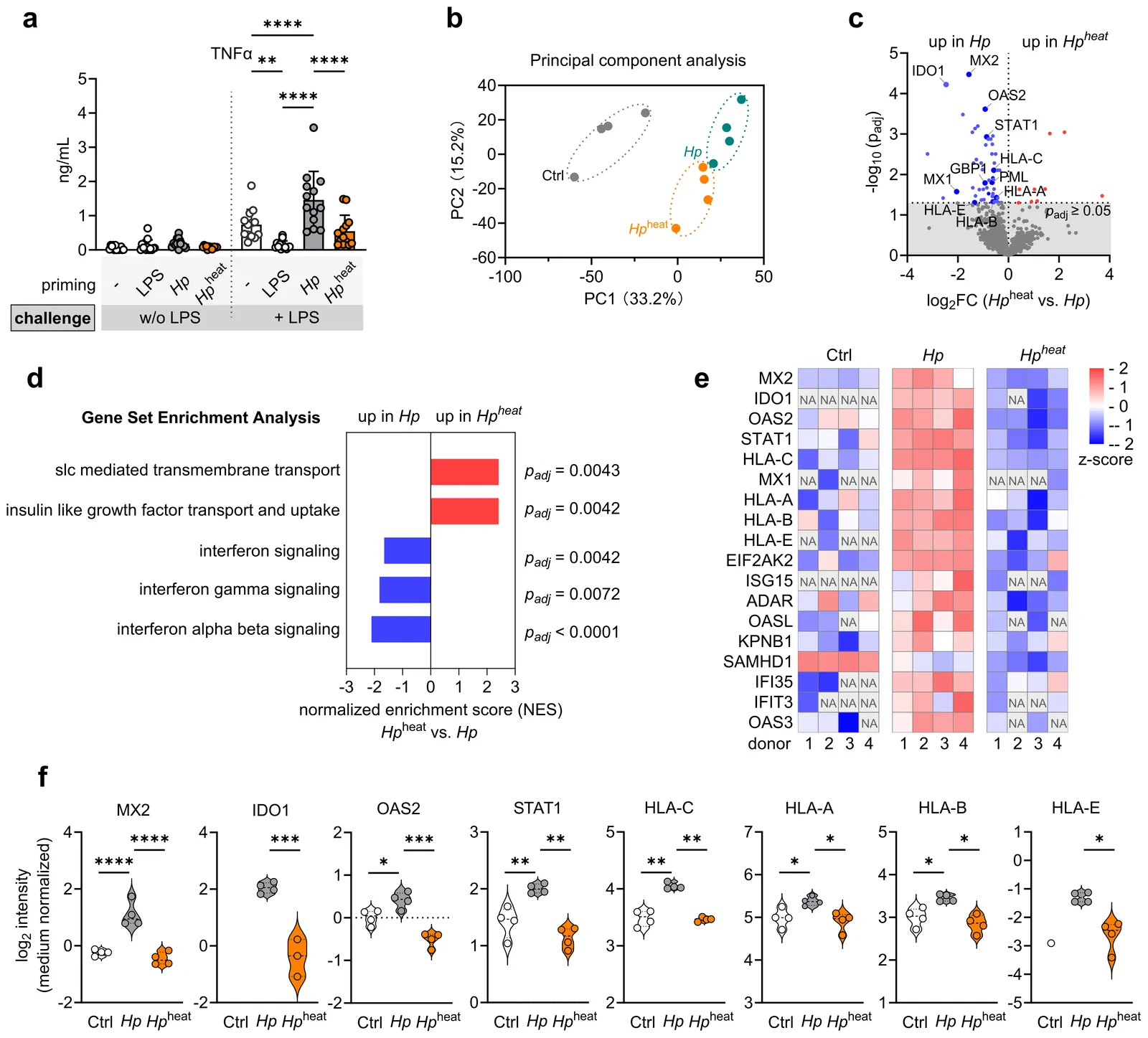
Viable *H. pylori* promotes interferon signaling activation in human primary monocytes. Monocytes were unprimed (“-”), or primed with LPS (5 ng/mL), viable *H. pylori* (*Hp)*, heat-killed *H. pylori* (*Hp*^heat^) (MOI 5) for 24 h. After medium refresh, cells were challenged without or with LPS (10 ng/mL) for 24 h. **(a)** TNF-α secretion was measured via ELISA. Data are presented as mean ± SD of 13 individual donors from at least two independent experiments. For statistical analysis, RM one-way ANOVA with Tukey’s *post-hoc* test was performed, p**** ≤ 0.0001 and p** ≤ 0.01. **(b-f)** Mass-spectrometry proteomic analysis of untreated control (Ctrl), *Hp- and Hp*^heat^ -primed monocytes (24 h) from four individual donors. **(b)** Principal component analysis of the proteomic results. **(c)** Volcano plot showing significantly up (red)- and downregulated (blue) proteins, *Hp*^heat^ compared to *Hp*. **(d)** Gene Set Enrichment Analysis showing the significantly upregulated and downregulated pathways, *Hp*^heat^ compared to *Hp*. **(e)** Heatmap showing z-scores of the normalized intensities of differentially expressed interferon signaling-associated proteins. NA: not available. **(f)** Box and violin plots showing the normalized intensities of selected differentially expressed proteins associated with interferon signaling. Boxes indicate the interquartile range (IDR) with the median shown as a center line; whiskers extend to the minimum and maximum values within 1.5 × IRQ. Violin plots illustrate the full distribution of the data. Normalized protein intensities (log2-tranformed and median-subtracted) are shown. For statistical analysis, differential protein abundance was analyzed using limma with a paired experimental design. Resulting *p* values were adjusted for multiple comparisons using the Benjamini–Hochberg FDR method, p**** ≤ 0.0001, p*** ≤ 0.001, p** ≤ 0.01 and p* ≤ 0.05.

In addition to impaired IFN signaling, *Hp^heat^*-treated monocytes exhibited reduced expression of pro-inflammatory cyclooxygenase (COX)-2, which is in line with previous findings showing that heat-inactivation of *H. pylori* decreased COX-2 expression in gastric epithelial cells (35), along with increased expression of the M2-associated markers early growth response 2 (EGR2) and mannose receptor C-type 1 (MRC1) (36) (**Supplementary Figures S2d, e**). Furthermore, viable *H. pylori*, but not *Hp*^heat^, induced mRNA expression of the COX-2 encoding gene prostaglandin endoperoxide synthase 2 (*PTGS2*), after 8 h, which was maintained until 24 h (**Supplementary Figure S2f**), resulting in elevated production of PGE_2_ during priming (**Supplementary Figure S2g**). PGE_2_ is classically considered a pro-inflammatory lipid mediator, and can additionally exert context-dependent immunoregulatory effects, including the initiation of anti-inflammatory responses (37) and regulation of innate immunity (38). We therefore hypothesized that the COX-2-PGE_2_ pathway contributes to viable *H. pylori*-induced hypersensitivity in our settings. However, neither pharmacological inhibition of COX-2 with celecoxib (39), which reduced PGE_2_ production (**Supplementary Figure S2g**), nor supplementation with exogenous PGE_2_ significantly altered TNF-α production following LPS challenge in *H. pylori* or *Hp*^heat^ -primed monocytes (**Supplementary Figure S2h**), arguing against a central role of PGE_2_ in this process.

Collectively, our results indicate that activation of IFN signaling is a key distinguishing feature between viable *H. pylori-* and *Hp^heat^*- treated monocytes and is accompanied by elevated COX-2 and PGE_2_ upon viable *H. pylori* stimulation. Yet, viable *H. pylori*-induced hypersensitivity is not mediated by the COX-2-PGE_2_ pathway, suggesting the involvement of IFN signaling or alternative mechanisms.

### Heat and PFA-inactivated, but not antibiotics-treated *H. pylori* failed to activate interferon signaling and to promote hypersensitivity to LPS

To explore how *H. pylori*-induced hypersensitivity and IFN signaling activation are influenced by bacterial physiological state, we compared *Hp^heat^*, characterized by thermal damage and loss of metabolic activity (40), with PFA-inactivated *H. pylori* (*Hp^PFA^*), which preserves bacterial structure despite metabolic inactivation (41), and antibiotic-treated *H. pylori* (*Hp^ab^*), which mimics physiological conditions under antimicrobial therapy (42). All preparations lost colony-forming capacity upon replating (**Supplementary Figure S3a**). Compared to viable *H. pylori*, TNF-α release upon subsequent LPS challenge was markedly reduced in monocytes exposed to *Hp^PFA^* during the priming phase, mirroring the response observed with *Hp^heat^* (**Figure 2a**). In contrast, *Hp^ab^-*priming, generated by exposure to an antibiotics cocktail containing amoxicillin and clarithromycin (42), potentiated hypersensitivity to LPS similar to viable *H. pylori* (**Figure 2b**). Although not culturable, antibiotic-treated bacteria may maintain near-native architecture (43) and remain largely metabolically active (44). These results indicate that the response to subsequent LPS challenge is strongly associated with bacterial physiological state: both viable *H. pylori and Hp^ab^* induce hypersensitivity, whereas *Hp^heat^* and *Hp^PFA^* elicit comparable impaired responses.

**Figure 2:**
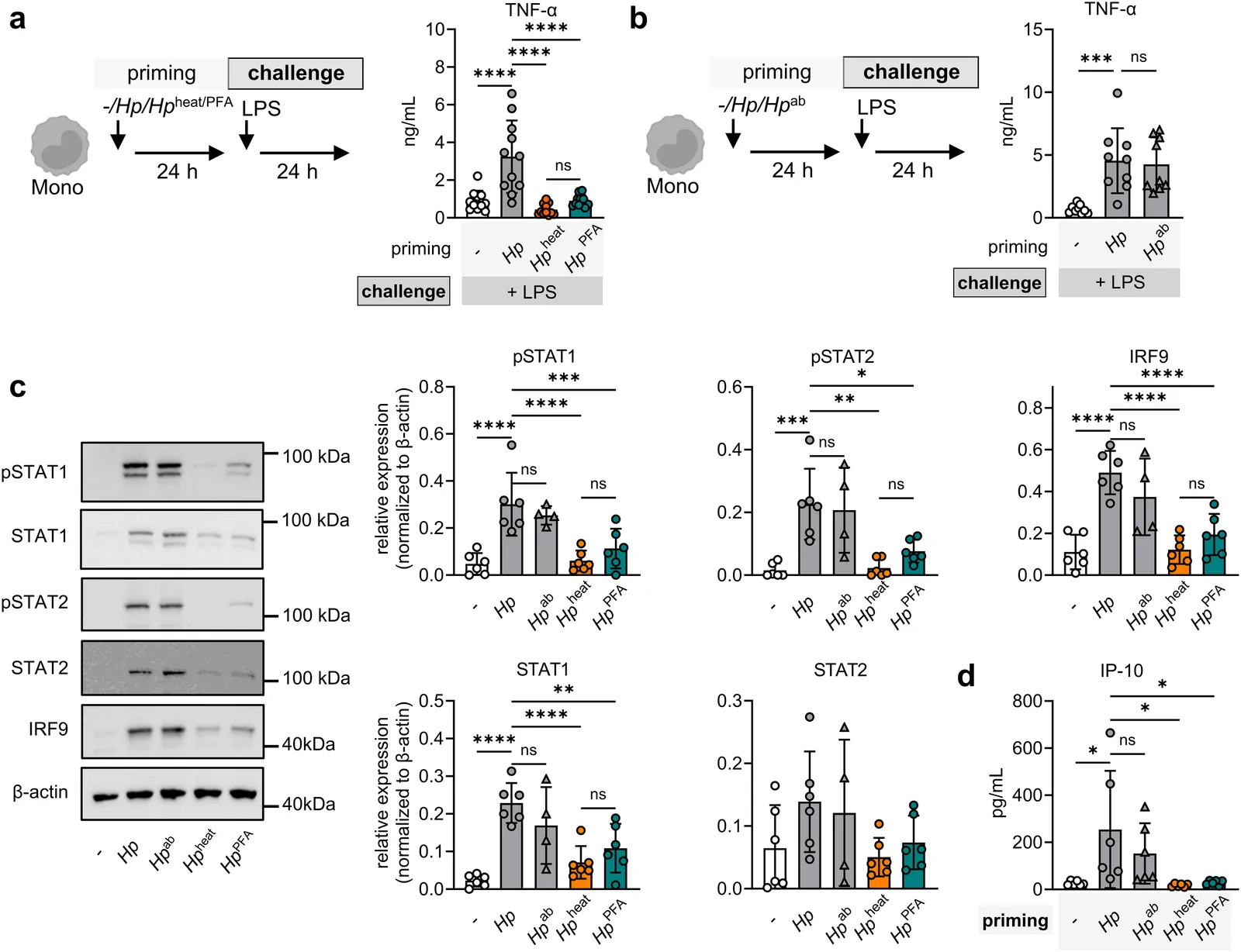
Heat and PFA-inactivated, but not antibiotics-treated *H. pylori* failed to activate interferon signaling and to promote hypersensitivity to LPS. Monocytes were unprimed (“-”), or primed with *H. pylori* (*Hp)*, heat-killed *H. pylori* (*Hp*^heat^), PFA-inactivated *H. pylori* (*Hp*^PFA^) or antibiotics-killed *H. pylori* (*Hp*^ab^) (MOI 5) for 24 h. After medium refresh, cells were challenged without or with LPS (10 ng/mL) for 24 h. **(a, b)** TNF-α secretion after challenge was measured via ELISA. **(c)** Protein expression of phosphorylated STAT1 (pSTAT1), STAT1, phosphorylated STAT2 (pSTAT2), STAT2 and IRF9 in monocytes after priming was measured by Western Blot, relative expression was normalized to β-actin. **(d)** IP-10 levels after priming were measured via ELISA. Data are presented as mean ± SD of **(a)** 11, **(b)** 9, **(c)** 4–6 or **(d)** 6 individual donors from at least two independent experiments. For statistical analysis, RM one-way ANOVA with Tukey’s *post-hoc* test was performed, p**** ≤ 0.0001, p*** ≤ 0.001, p** ≤ 0.01 and p* ≤ 0.05; ns: not significant.

Next, we investigated how these conditions (*Hp^heat^, Hp^PFA^ and Hp^ab^*) affect type I IFN and type II IFN signaling by analyzing phosphorylated STAT1 (pSTAT1), a key mediator of type II IFN responses, as well as phosphorylated STAT2 (pSTAT2) and IRF9, which together with pSTAT1 form the ISGF3 complex that drives type I IFN-stimulated gene expression (1). Viable *H. pylori* induced STAT1 and STAT2 phosphorylation as well as total STAT1 and STAT2 and increased IRF9 expression (**Figure 2c**). In contrast, *Hp^heat^* or *Hp^PFA^* failed to activate these signaling components, whereas *Hp^ab^* retained this capacity (**Figure 2c**). A comparable pattern was recognized for IP-10, an IFN induced cytokine, further corroborating the activation of IFN signaling in *Hp^ab^* but not *Hp^heat^* and *Hp^PFA^* (45) (**Figure 2d**).

We further examined the expression of type I IFN and type II IFN, related signaling components and IFN responsible genes. Viable *H. pylori* induced expression of type I IFN genes IFNA2 and IFNB1 as well as the type II IFN gene IFNG, whereas *Hp^heat^* or *Hp^PFA^* failed to induce these transcripts within a timeframe of 1 h to 24 h (**Supplementary Figure S3b**). Activation of upstream regulators IRF3 and IRF7, STAT1, and downstream IFN-responsive genes such as ISG15, IDO1 as well as suppressors of cytokine signaling (SOCS) -1, -2 and -3, was evitable only in viable *H. pylori-*primed monocytes (**Supplementary Figure S3c**).

Together, these data demonstrate that heat and PFA inactivation abolish the ability of *H. pylori* to activate IFN signaling and induce hypersensitivity to subsequent LPS, whereas this capacity is preserved following antibiotic treatment.

### Interferon signaling during challenge is dispensable for *H. pylori*-induced hypersensitivity

IFN signaling plays a crucial role in regulating inflammatory responses (9) and has emerged as a key regulator of immune priming and memory, orchestrating both priming and retaining the balance between training and tolerance (10). Given the marked differences between viable and heat/PFA-inactivated *H. pylori* in both, IFN signaling activation during priming and hypersensitivity upon subsequent LPS challenge, we hypothesized that type I IFN and/or type II IFN signaling is required for the hypersensitivity induced by viable bacteria.

To delineate the contributions of type I IFN and type II IFN signaling, cells were stimulated with IFN-α or IFN-β (type I) or IFN-γ (type II), and the impact of IFNAR blockade was evaluated using anifrolumab. The general efficacy of IFN pathway modulation was confirmed by measuring IP-10, a marker of IFN signaling (45), which was increased upon treatment with type I IFNs or IFN- γ (**Supplementary Figures S4a-c**) and decreased in the presence of anifrolumab (**Supplementary Figure S4d**).

As priming monocytes with viable *H. pylori* results in enhanced IFN signaling which was not observed under *Hp^heat^* or *Hp^PFA^* conditions, we first examined whether impaired hypersensitivity in *Hp^heat^* or *Hp^PFA^*-primed monocytes results from insufficient IFN levels during the challenge phase. However, manipulation of type I IFN signaling during the challenge phase, either by supplementation with IFN-α and IFN-β or by IFNAR blockade using anifrolumab, neither restored TNF-α induction in *Hp^heat^* or *Hp^PFA^*-primed monocytes nor inhibited the elevated TNF-α response in viable *H. pylori*-primed cells (**Figure 3a**). Similarly, exogenous IFN-γ did not rescue the impaired TNF-α response in *Hp^heat^* or *Hp^PFA^* conditions (**Figure 3b**). Based on these findings, we excluded that type I IFN or type II IFN signaling can directly stimulate LPS-induced TNF-α production during challenge. This is consistent with reports showing that TNF-α production by LPS in macrophages does not depend on IFN-β or IFNAR signaling (46). Moreover, this is further supported by our results that modulation of IFN signaling during the challenge phase did not alter basal TNF-α production in the absence of subsequent LPS stimulation (**Supplementary Figure S4e**).

**Figure 3:**
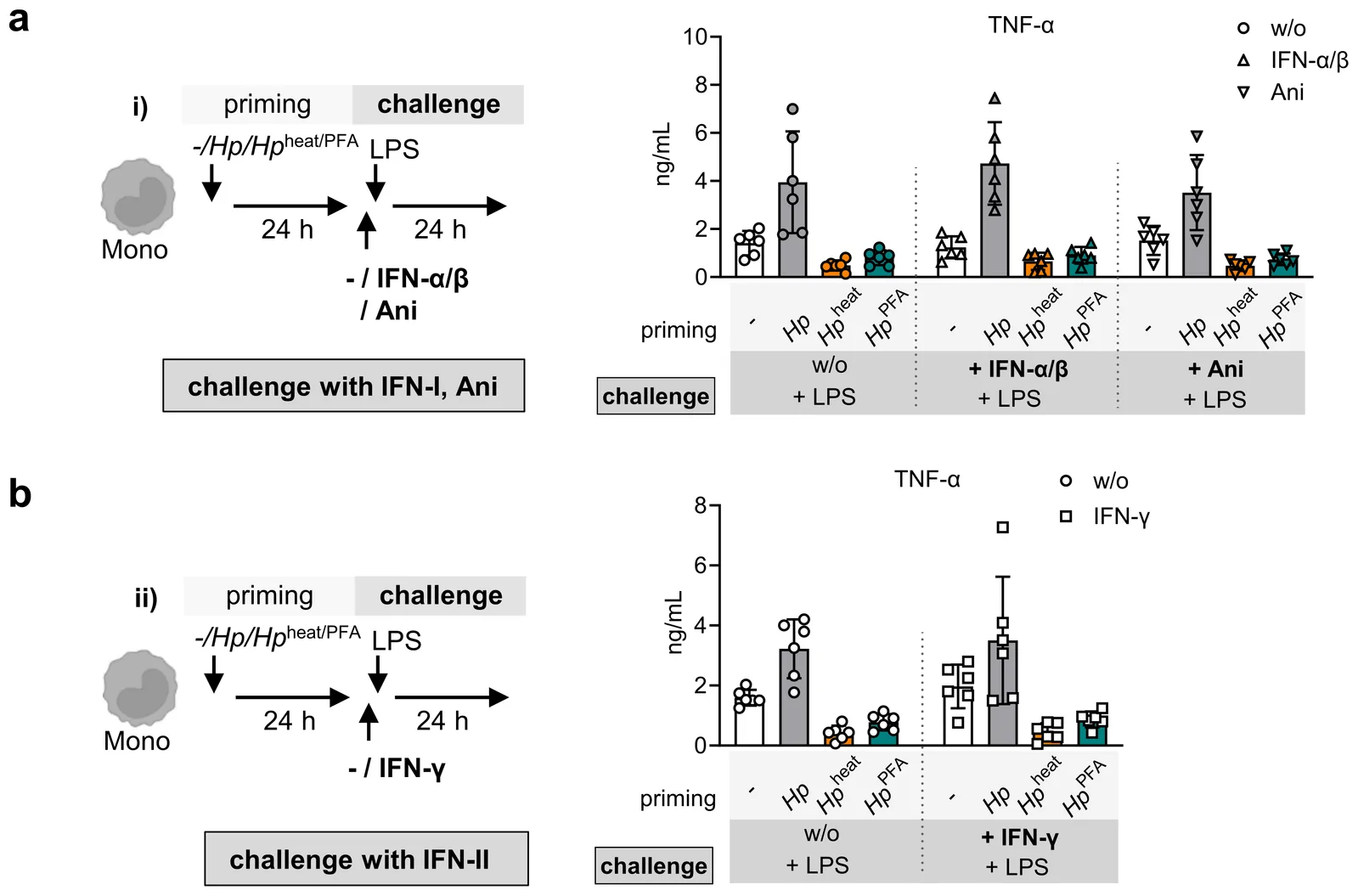
Modulating interferon signaling during challenge does not impact *H. pylori*-induced hypersensitivity to LPS. Monocytes were unprimed (“-”), or primed with viable *H. pylori* (*Hp)*, heat-killed *Hp* (*Hp*^heat^), PFA-inactivated *Hp* (*Hp*^PFA^) (MOI 5) for 24 h. After medium refresh, cells were treated without (w/o) or with **(a)** IFN-α, IFN-β (10 ng/mL each), or the interferon-α/β receptor (IFNAR) antagonist anifrolumab (20 ng/mL), or **(b)** IFN-γ (20 ng/mL) for 15 min, and then challenged without or with LPS (10 ng/mL) for 24 h. TNF-α secretion after challenge was measured via ELISA. Data are presented as mean ± SD of 6 individual donors from at least two independent experiments.

Altogether, these results indicate that activation of type I IFN or type II IFN signaling during the challenge phase is dispensable for hypersensitivity to LPS challenge induced by viable *H. pylori*.

### *H. pylori*-induced hypersensitivity requires type I interferon signaling during priming

Type I IFNs and type II IFN are well-established drivers of epigenetic memory that confer faster, stronger and more stable responses upon restimulation (4, 47). We next investigated whether manipulating type I IFN and/or type II IFN signaling during priming shapes the subsequent response to LPS challenge.

Notably, modulation of type I IFN or type II IFN signaling barely impacted TNF-α production during the priming phase (**Supplementary Figures S5a-c**), except a minor reduction in *H. pylori*-induced TNF-α by IFN-α and IFN-β priming (**Supplementary Figure S5a**). Of interest, activation or blockade of type I IFN signaling did not alter basal TNF-α levels during the challenge phase in the absence of LPS, but markedly influenced the response to subsequent LPS challenge (**Figures 4a, b**). As shown in **Figure 4a**, addition of IFN-α and IFN-β did not alter the TNF-α production in unprimed monocytes or those infected with viable *H. pylori*, but restored the impaired TNF-α response in *Hp^PFA^*-treated, and to a lesser extent, in *Hp^heat^*-treated monocytes, whereas blockade of type I IFN signaling with anifrolumab largely abolished the elevated TNF-α production induced by viable *H. pylori* (**Figure 4b**). In contrast, priming monocytes with IFN-γ alone did not elicit similar responses (**Figure 4c**), indicating that activation of type II IFN signaling alone is insufficient to induce viable *H. pylori*-induced hypersensitivity. When monocytes were primed with both type I IFNs and type II IFN, the impaired TNF-α response in *Hp^PFA^*-treated monocytes was similarly restored, along with a weaker effect in unprimed and *Hp^heat^*-treated ones, resembling the effects of type I IFN priming alone (**Figure 4d**). Collectively, these findings reveal a critical role of type I IFN, rather than type II IFN signaling during priming in the viable *H. pylori*-induced hypersensitivity to subsequent LPS.

**Figure 4:**
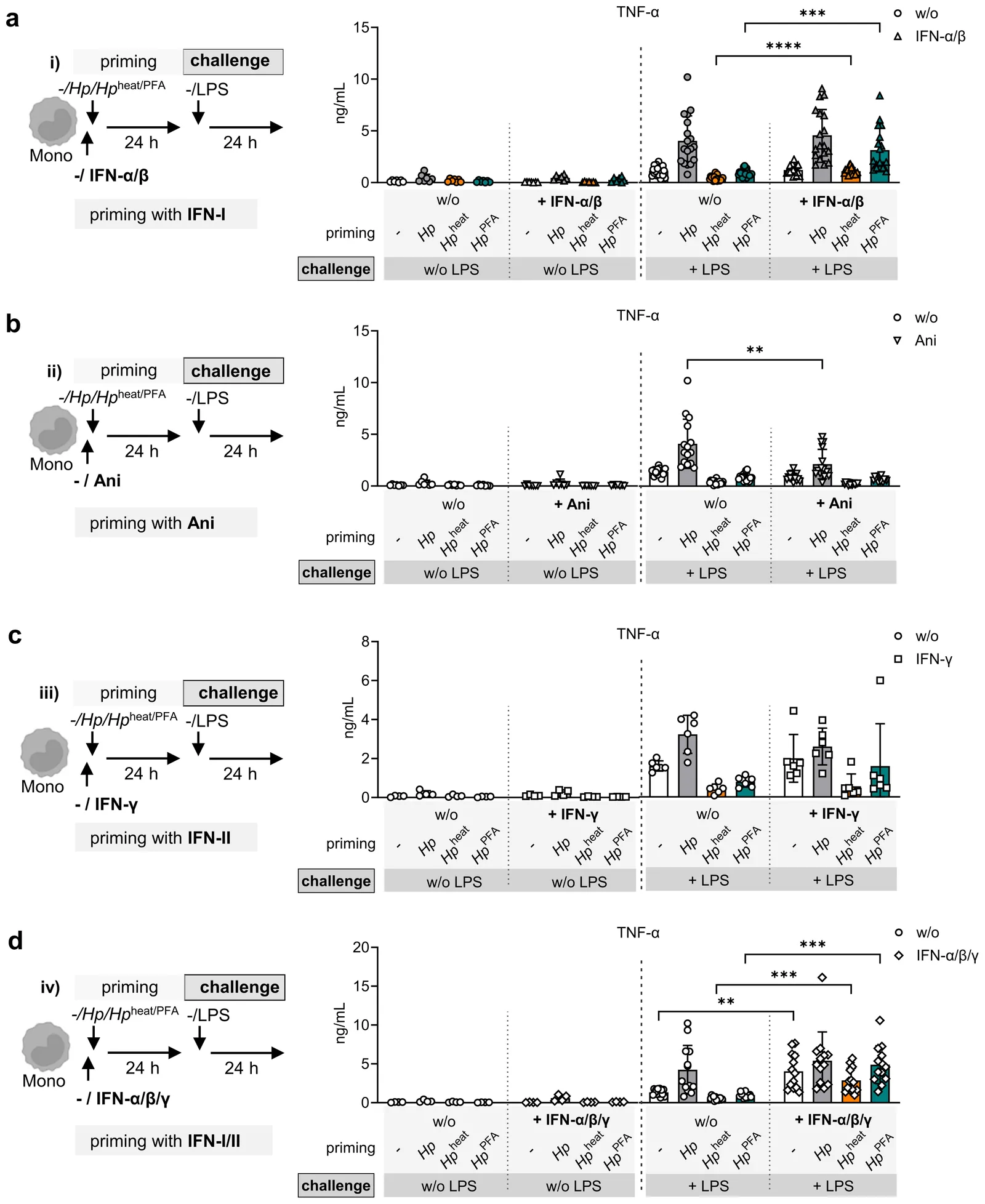
*H. pylori*-mediated hypersensitivity to LPS requires type I, but not type II interferon signaling during priming. Monocytes were pre-treated without (w/o), or with a-b) IFN-α, IFN-β (10 ng/mL each), the interferon-α/β receptor (IFNAR) antagonist anifrolumab (20 ng/mL) or **c-d)** IFN-γ (20 ng/mL) for 15 min, then unprimed (“-”) or primed with viable *H. pylori (Hp)*, heat-killed *H. pylori* (*Hp*^heat^), PFA-inactivated *H. pylori* (*Hp*^PFA^) (MOI 5) for 24 h. After medium refresh, cells were challenged without (w/o) or with LPS (10 ng/mL) for 24 h. TNF-α secretion after **(a, c)** priming or **(b, d)** challenge was measured via ELISA. Data are presented as mean ± SD of **a)** 7 (w/o LPS), 18 (+ LPS); **(b)** 7 (w/o LPS), 15 (+ LPS); **(c)** 4 (w/o LPS), 6 (+ LPS); or **(d)** 4 (w/o LPS), 13 (+ LPS) individual donors from at least two independent experiments. For statistical analysis, RM two-way ANOVA with Šídák’s *post-hoc* test was performed; p**** ≤ 0.0001, p*** ≤ 0.001 and p** ≤ 0.01.

### Additional bacteria-associated factors contribute to *H. pylori*-induced hypersensitivity

The weaker impact of type I IFN priming on *Hp^heat^* compared to *Hp^PFA^* suggests the involvement of alternative mechanisms (38). To further investigate the role of viable *H. pylori* in the context of hypersensitivity, we combined *Hp^heat^* and viable *H. pylori* treatment and investigated how the presence of *Hp^heat^* impacts *H. pylori*-induced hypersensitivity and IFN signaling activation. Compared to viable *H. pylori* alone, monocytes primed with both *Hp^heat^* and viable *H. pylori* showed reduced TNF-α production during the priming phase (**Figure 5a**) and abolished the hypersensitivity to subsequent LPS challenge (**Figure 5b**). This is accompanied by a partial inhibition on key IFN signaling components, including phosphorylated STAT1, phosphorylated STAT2, total STAT1 and STAT2 as well as IRF9, without affecting STAT3 phosphorylation or expression of total STAT3, indicating a specific effect on type I IFN signaling (**Figure 5c**). Similarly, co-treatment with *Hp^heat^* also inhibited IP-10 production induced by viable *H. pylori* (**Figure 5d**). These results indicate an inhibitory role of *Hp^heat^* on the activation of type I IFN signaling induced by viable *H. pylori* during priming, thereby abolishing the hypersensitivity response to subsequent LPS. In line with this, priming with type I IFNs restored the impaired hypersensitivity in monocytes exposed to the combination of *Hp^heat^* and viable *H. pylori*, an effect that is much stronger compared to those treated with *Hp^heat^* alone, where only weak restoration was evitable (**Figure 5e**). These findings further support a critical role for type I IFN signaling in *H. pylori*-induced hypersensitivity but also indicate that exogenous IFN alone failed to restore the effects of *Hp^heat^* in the absence of viable bacteria. Conversely, *Hp^heat^* was able to attenuate the response induced by viable *H. pylori* under conditions lacking IFN signaling. Together, these observations suggest that multiple factors associated with viable *H. pylori* and/or *Hp^PFA^*- cooperate with type I IFN signaling to drive the full hypersensitivity response.

**Figure 5:**
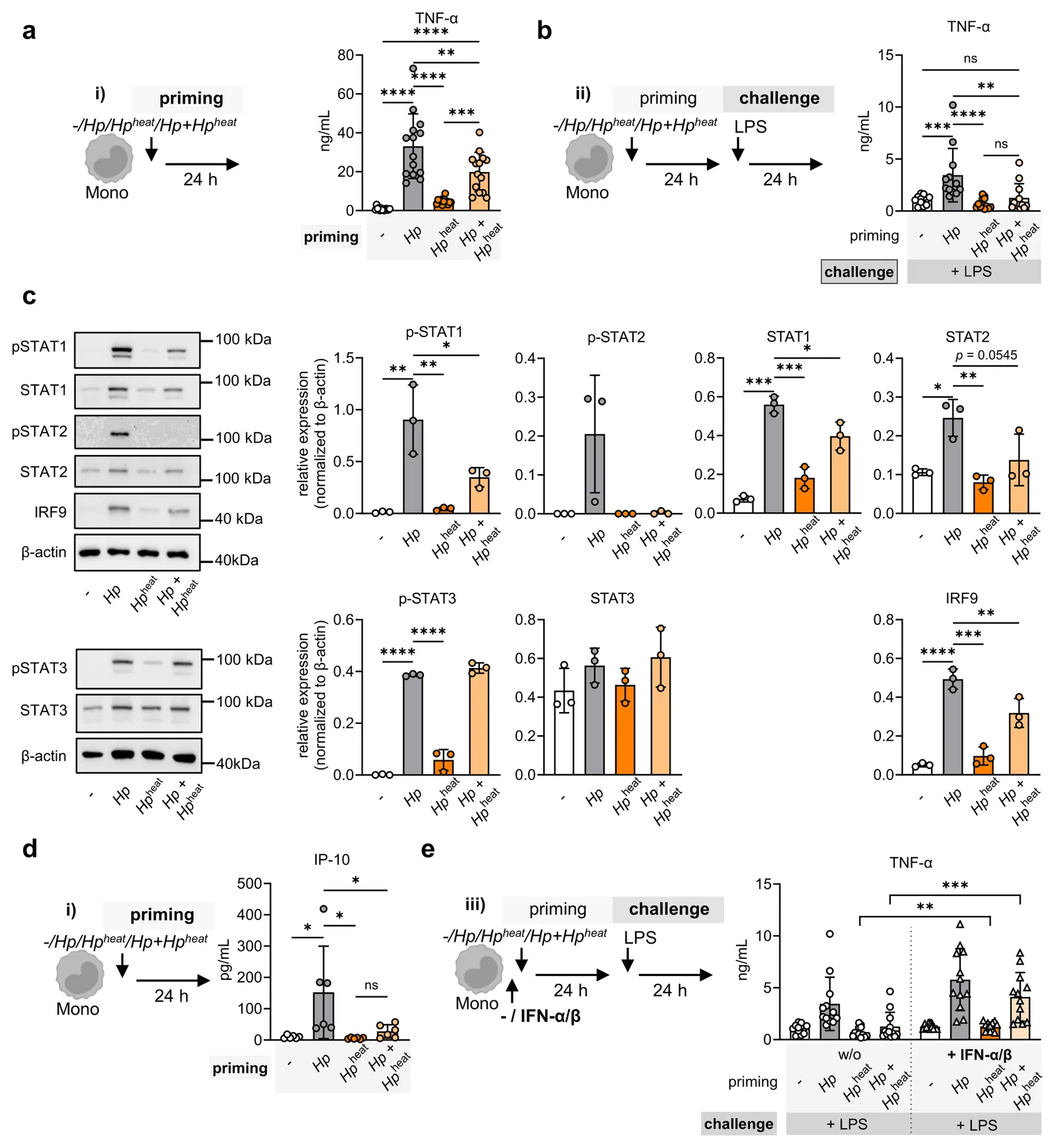
Heat-killed *H. pylori* partially inhibits the ability of viable *H. pylori* to activate type I interferon signaling. Monocytes were left unprimed (“-”) or primed with viable *H. pylori (Hp)*, heat-killed *H. pylori* (*Hp*_heat_), or the combination of *Hp* and *Hp*_heat_ (MOI 5) for 24 h. After medium refresh, cells were challenged without or with LPS (10 ng/mL) for 24 h. TNF-α levels after **(a)** priming or **(b)** challenge and **(d)** IP-10 levels after priming were measured via ELISA. **(c)** Protein expression of p-STAT1, p-STAT2, IRF9, STAT1, STAT2, p-STAT3 and STAT3 in monocytes after priming was measured by Western Blot, relative expression was normalized to β-actin. **(e)** Monocytes were pre-treated without (w/o) or with IFN-α, IFN-β (10 ng/mL each) for 15 min prior to priming and challenge with indicated stimuli. TNF-α levels were monitored after challenge. Data are presented as mean ± SD of **(a)** 14, **(b, e)** 12; **(c)** 3; **(d)** 6, or **(e)** 12 individual donors from at least two independent experiments. For statistical analysis, **(a–d)** RM one-way with Tukey’s post-hoc test or **(e)** RM two-way ANOVA with Šídák's post-hoc test was performed; p**** ≤ 0.0001, p*** ≤ 0.001, p** ≤ 0.01 and p* ≤ 0.05.

## Discussion

Our previous work demonstrated that *Hp* induces immune priming characterized by enhanced responses to subsequent LPS challenge (18). In the present study, we show that activation of IFN signaling is a key feature distinguishing monocytes exposed to viable *H. pylori* from those treated with heat- or PFA-inactivated bacteria. Furthermore, we identify type I IFN signaling as a critical determinant of *H. pylori*-induced inflammatory hypersensitivity.

*Hp* infection triggers a broad range of innate immune responses, including the production of pro-inflammatory cytokines, chemokines (18) and lipid mediators such as PGE_2_ (35, 48). Although our proteomic analysis revealed strong upregulation of COX-2 and increased PGE_2_ production in monocytes infected with viable *H. pylori*, functional perturbation experiments showed that PGE_2_ suppresses rather than enhances LPS-induced TNF-α response. This is consistent with a previous study demonstrating the inhibitory effects of PGE_2_ on stimulation of secondary inflammation (49), supporting the immunosuppressive roles of PGE_2_ (50). These findings argue against a major role of the COX-2-PGE_2_ axis in *H. pylori*-induced hypersensitivity, and instead directed our focus toward IFN signaling, which is the most prominent pathway distinguishing monocytes infected with viable *H. pylori* from cells infected with heat-killed bacteria.

Besides viral infections, bacterial infections are also well-established activators of type I IFN and type II IFN signaling (1). Viable *H. pylori*, but not heat-killed or PFA-inactivated bacteria, induced a coordinated type I IFN and type II IFN response, including upstream regulators, canonical JAK–STAT signaling components, and downstream ISGs. Notably, antibiotic-treated *H. pylori* retained both IFN-activating capacity and the ability to enhance subsequent LPS responses, supporting a link between IFN signaling activation during the priming phase and the acquisition of inflammatory hypersensitivity. The preservation of IFN signaling following antibiotic treatment may reflect retention of bacterial structural integrity and viability-associated molecular patterns, which serve as PAMPs that are recognized by PRR to induce IFN responses (1). In addition, antimicrobial stress may drive *H. pylori* into a viable but non-culturable state (43), in which bacteria retain immunostimulatory capacity despite loss of cultivability (44). Antibiotic treatment may also promote the release of bacterial DNA, which has been shown to amplify inflammatory responses via TLR9 activation (51), thereby contributing to the sustained IFN signaling and inflammatory priming.

IFNs are increasingly recognized as central regulators of inflammatory adaptation (9) and immune priming and memory (10). Our short-term immune priming model is consistent with the “training-priming continuum” concept (10), in which IFN activity may persist after initial stimulation and contributes to durable cellular programming. Accordingly, we considered two non-mutually exclusive hypotheses: 1) sustained IFN signaling at the time of secondary challenge synergizes with LPS signaling to amplify inflammatory responses; 2) IFN signaling during priming programs monocytes for enhanced inflammatory hypersensitivity upon secondary stimuli.

Diverse studies have highlighted the complex and context-dependent interactions between IFN signaling and LPS-induced inflammatory response (52). Although LPS activates both, the MyD88-dependent NF-κB pathway, which drives pro-inflammatory cytokine production, and the TRIF-dependent pathway, which induces IRF3 activation and type I IFN signaling (53), TNF-α production is largely independent of type I IFN signaling (46). IFN-γ can directly enhance TNF-α transcription (54), and stabilize LPS-induced TNF-α mRNA and protein expression (55), illustrating a more direct synergistic interaction between IFN-γ and LPS-TLR4 signaling. However, in our system, neither type I IFNs nor type II IFN directly induced TNF-α production either in the absence or presence of LPS. Thus, these findings suggest that IFN activity during the secondary stimulation phase is not a major driver of *H. pylori*-induced hypersensitivity.

Instead, our data suggest that type I IFN signaling during the priming phase establishes a reprogrammed state that determines subsequent responsiveness to LPS, consistent with the concept of a “training-priming continuum” of immune priming. In line with this, modulation of type I, but not type II, IFN signaling during *H. pylori* priming critically shaped the subsequent inflammatory response to LPS. This differs from previous work showing that priming with IFN-γ, but not IFN-β enhanced LPS responsiveness in monocytes (56). These discrepancies likely reflect differences in experimental contexts: including the absence of concurrent *H. pylori*-induced pattern-recognition and inflammatory pathways in the study from Yu et al. Additionally, culture conditions such as macrophage colony-stimulating factor exposure in their system may further influence monocyte differentiation (57), IFN responsiveness as well as response to secondary stimuli (58). Consequently, differences in cellular state may alter the relative contribution of type I and type II IFN pathways to subsequent LPS responses. In a context-dependent manner, various studies have reported epigenetic reprogramming by type I (12–15, 59) and type II IFN signaling (60–62) to promote inflammatory responses.

The differential contribution of type I IFNs and type II IFN to *H. pylori*-induced hypersensitivity may also reflect their distinct biological origins. Type I IFNs are induced directly downstream of pattern-recognition receptor signaling and represent an intrinsic component of the monocyte response to microbial sensing (2). In contrast, IFN-γ is predominantly produced by lymphoid cells, such as NK cells and T lymphocytes, and therefore mediating communication between innate and adaptive immune compartments (63). In our monocyte-based system, the absence of physiological cellular sources of IFN signals likely limits the contribution of type II IFN signaling. Under these conditions, type I IFN signaling may represent the dominant IFN pathway involved in establishing inflammatory reprogramming, whereas the contribution of type II IFN signaling may become more prominent in the context of multicellular immune interactions.

Notably, exogenous type I IFNs alone did not reproduce the *H. pylori*-induced hypersensitivity, indicating that IFN signaling is insufficient to drive the response independently. While exogenous type I IFNs restored hypersensitivity in *Hp^PFA^*-treated monocytes, their effects were considerably weaker in *Hp^heat^*-treated cells, suggesting that additional bacterial factors contribute to full establishment of the hyperresponsive state. Moreover, *Hp^heat^* inhibits the induction of IFN signaling induced by viable *H. pylori* in monocytes co-treated with both, consequently simultaneous treatment with *Hp^heat^* and viable *H. pylori* failed to induce hypersensitivity. Yet, while exogenous type I IFNs failed to restore hypersensitivity in *Hp^heat^* treated monocytes, it restored the responsive state upon treatment with *Hp^heat^* and viable *H. pylori*. This indicates that *Hp^heat^* fails to induce a yet unknown signaling axis that is a key driver of the hyperresponsive state in monocytes and it actively suppresses the induction of type I IFN signaling, which viable *H. pylori* cannot overcome. Thus, the inability of *Hp^heat^* to induce hypersensitivity to LPS results cannot be explained solely by its impaired induction of type I IFN responses, as this inhibitory effect is retained even in the presence of viable *H. pylori*. Heat inactivation also seems to destroy or mask bacterial factors that are required to promote the enhanced responsiveness induced by viable *H. pylori*. Together, these findings indicate that *H. pylori*-induced hypersensitivity requires integration of type I IFN signaling with additional bacterial factors, that are preserved in viable *H. pylori* and *Hp^PFA^*, but altered or lost upon heat inactivation.

Finally, our findings may have broader implications for chronic inflammatory and autoimmune diseases. Although type I IFNs are essential mediators of host defense (1), sustained or excessive type I IFN signaling can promote pathological inflammation (9). In support of this, endogenous IFN-β has been shown to amplify inflammatory cytokine production in response to LPS, and IFN-β-deficient mice exhibit reduced pro-inflammatory cytokine levels and improved survival in an *in vivo* model using LPS-induced lethality (64). Clinically, persistent *H. pylori* infection has been associated with an increased risk of autoimmune diseases like systemic lupus erythematosus (65–67), a prototypic type I IFN-driven autoimmune disease (68). Although the causal relationship between *H. pylori* infection and systemic lupus erythematosus remains unproven, our findings raise the possibility that chronic exposure to *H. pylori* may induce a type I IFN activation state that primes monocytes toward exaggerated inflammatory responses to secondary stimuli. Thus, type I IFN signaling exhibits context-dependent pathophysiological roles, with beneficial roles in antimicrobial defense (69), but potential pathological consequences when persistently activated, which is the case for *H. pylori* infection.

Our findings are derived from primary human monocytes, which model the early wave of infiltrating myeloid cells recruited during mucosal infection (70). Whether this type I IFN-dependent priming is preserved upon macrophage differentiation remains an open question. Notably, in parallel experiments using monocyte-derived macrophages, *H. pylori* failed to induce enhanced TNF-α production upon LPS challenge (data not shown), suggesting that the hyperresponsive phenotype is cell-specific. Therefore, our conclusions are most relevant to circulating or recently recruited monocytes, rather than to tissue-resident macrophages that have undergone terminal differentiation.

In conclusion, our study identifies type I IFN signaling as a key mechanism underlying the viability-dependent inflammatory hypersensitivity induced by *H. pylori* in human monocytes. These findings reveal a previously unappreciated role of type I IFN signaling in immune priming and memory, advance the understanding of host-pathogen interactions and provide new insights into how *H. pylori* drives long-term inflammatory responses.

### Limitation of the study

Our current study identifies type I IFN signaling as an essential and critical mediator of *H. pylori*-induced hypersensitivity, yet the downstream molecular events and bacterial factors involved, remain to be fully elucidated. Although we did not detect viable bacteria after the challenge phase, we cannot exclude that minor residual bacterial factors persist in the culture after priming and might contribute to the LPS hypersensitivity.

## Data Availability

The datasets presented in this study can be found in online repositories. The names of the repository/repositories and accession number(s) can be found below: http://www.proteomexchange.org/, PXD080861.

## Glossary

APOA1: Apolipoprotein A1
COX: Cyclooxygenase
EGR2: Early growth response 2
HLA: Human leukocyte antigen
H. pylori: Helicobacter pylori
Hpab: Antibiotic-treated H. pylori
Hpheat: Heat-killed H. pylori
HpPFA: PFA-inactivated H. pylori
HPLC: High-performance liquid chromatography
HPLC-MS/MS: High-performance liquid chromatography–tandem mass spectrometry
IDO: Indoleamine 2, 3-dioxygenase
IFN: interferon
IFNAR: Interferon-α/β
IP-10: Interferon gamma-induced protein 10
ISG: Interferon-stimulated genes
ISGF3: Interferon-stimulated gene factor 3
JAK: Janus kinase
LPS: Lipopolysaccharide
MRC1: Mannose receptor C-type 1
MX2: MX dynamin like GTPase 2
NF-кB: Nuclear factor к
OAS2: 2’-5’-oligoadenylate synthetase 2
PAMP: Pathogen-associated molecular patterns
PBMC: Peripheral blood mononuclear cells
PRR: Pattern-recognition receptors
pSTAT1: Phosphorylated STAT1
pSTAT2: Phosphorylated STAT2
PTGS2: Prostaglandin endoperoxide synthase 2
SOCS: Suppressors of cytokine signaling
STAT: Signal transducer and activator of transcription
TNF-α: Tumor necrosis factor alpha
