# Supplementary material for: Helicobacter pylori-induced immune priming requires type I interferon signaling
Source: Front Immunol. 2026 Oct 2;17:1942802. doi: 10.3389/fimmu.2026.1942802 (PMC13672020; doi:10.3389/fimmu.2026.1942802)
Supplement: Supplementary file 1 [file SupplementaryFile1.docx]

**Supplementary Figures**

**Fig.S1 Assessment of viable *H. pylori* in monocyte supernatants after priming and after challenge**

Monocytes were primed in monocyte medium with viable *H. pylori* (MOI 5) for 24 h. Extracellular bacteria were removed by washing with monocyte medium with penicillin/streptomycin (P/S), and monocytes were further incubated in monocyte medium with P/S for another 24 h. Supernatants were collected after **a)** priming and **b)** after the subsequent incubation phase (challenge), and plated on GC agar plates supplemented with 10% horse serum under microaerophilic conditions at 37°C for 6 days. Colony formation was monitored by photography, 2 representative donors from n = 3 were shown.

**Fig.S2 Differentially regulated proteins between heat-killed and viable *H. pylori***

**a-f)** Monocytes were untreated (Ctrl), or treated with viable *H. pylori* (*Hp) or* heat-killed *H. pylori* (*Hp*^heat^) (MOI 5) for **a-e)** 24 h or **f)** 1-24 h. **a-e)** Mass-spectrometry proteomics analysis of untreated controls (“Ctrl”), *Hp- and Hp*^heat^ -primed monocytes (24 h) from four individual donors. **a-b)** Heatmap showing z-scores of the normalized intensities of differentially expressed proteins associated with **a)** regulation of insulin like growth factor transport and uptake or **b)** slc mediated transmembrane transport. NA: not available. **c, e)** Exemplary **b**ox and violin plots showing the normalized intensities of C3, APOA1, COX-2, EGR2 and MRC1. **d)** Volcano plot showing significantly up (red)- and downregulated (blue) proteins, *Hp*^heat^ compared to *Hp*. For statistical analysis, differential protein abundance was analyzed using limma with a paired experimental design. Resulting *p* values were adjusted for multiple comparisons using the Benjamini–Hochberg FDR method; p*** ≤ 0.001, p** ≤ 0.01 and p* ≤ 0.05. **f)** Relative mRNA expression of PTGS2 was measured by PCR, normalized to ARP. Data are presented as mean + SD from 3 individual donors from at least two independent experiments. For statistical analysis, RM two-way ANOVA with Tukey’s post-hoc test was performed. p**** ≤ 0.0001 and p** ≤ 0.01. **g-h)** Monocytes were pre-treated without (w/o) or with COX-2 inhibitor celecoxib (Cele, **g**: 10 µM, **h**: 1-10 µM) or supplemented with exogenous PGE_2_ (1-10 nM) for 15 min, and then unprimed (“-”), or primed with *Hp* or *Hp*^heat^ (MOI 5) for 24 h. After medium refresh, cells were challenged without or with LPS (10 ng/mL) for 24 h. **g)** PGE_2_ levels after priming were analyzed by HPLC-MS/MS, nd: not detectable (≤ LOD of 0.2 ng/mL). **h)** TNF-α levels after challenge was measured via ELISA. Data are presented as mean ± SD of **g)** 4 or **h)** 4-5 individual donors from at least two independent experiments. For statistical analysis, **g)** RM one-way or **h)** mixed -effects two-way ANOVA with Tukey’s post-hoc test was performed. p**** ≤ 0.0001 and p** ≤ 0.01.

**Fig.S3 Heat and PFA-inactivated *H. pylori* fail to activate interferon associated genes**

**a)** Exemplary pictures of viable *H. pylori* (*Hp*), heat-killed *H. pylori* (*Hp*^heat^), PFA-inactivated *H. pylori* (*Hp*^PFA^) or antibiotic-treated *H. pylori* (*Hp*^ab^) that are re-plated onto GC agar-plates and incubated for 72 h. **b-c)** Monocytes were untreated (Ctrl), or treated with *Hp*, *Hp*^heat^ or *Hp*^PFA^ (MOI 5) for **b)** 1-24 h or **c)** 24 h. mRNA expression of interferon responsive genes was measured by qPCR, relative expression was normalized to ARP. **b)** mRNA kinetics of IFNA2, IFNB1 and IFNG. **c)** Heatmap showing z-scores of the relative expression of STAT1, ISG15, IDO1, IRF3, IRF7, SOCS1, SOCS2 and SOCS3. Data are presented as mean ± SD of 3 individual donors from at least two independent experiments. For statistical analysis, RM two-way ANOVA with Tukey’s post-hoc test was performed, *p*** ≤ 0.01 and *p** ≤ 0.05.

**Fig.S4 Targeting type I & type II interferon signaling modulates IP-10 but not basal TNF-α production**

**a-d)** Monocytes were unprimed (“-”) or primed with **a)** IFN-α/IFN-β (10 ng/mL each), **b)** IFN-γ (20 ng/mL) **c)** IFN-α/IFN-β (10 ng/mL each) and IFN-γ (20 ng/mL) or **d)** IFN-α/IFN-β (10 ng/mL each) in the absence or presence of interferon-α/β receptor (IFNAR) antagonist anifrolumab (0.016 - 10 ng/mL) for 24 h. IP-10 secretion was measured via ELISA. Data are presented as mean ± SD of **a)** 12 **b)** 9 **c-d)** 6 individual donors from at least two independent experiments. For statistical analysis, **a-c)** two-tailed paired *t*-test, **d)** RM one-way ANOVA with Tukey’s post-hoc test and was performed, *p***** ≤ 0.0001, *p**** ≤ 0.001 and *p*** ≤ 0.01. **e)** Monocytes were unprimed (“-”), or primed with viable *H. pylori* (*Hp)*, heat-killed *H. pylori* (*Hp*^heat^), PFA-inactivated *H. pylori* (*Hp*^PFA^) (MOI 5) for 24 h. After medium refresh, cells were pre-treated with IFN-α, IFN-β (10 ng/mL each), anifrolumab, or IFN-γ (20 ng/mL) for 15 min, and then incubated in the absence of (w/o) LPS for 24 h. TNF-α secretion after challenge was measured via ELISA. Data are presented as mean ± SD of 4 individual donors from at least two independent experiments.

**Fig.S5 Impact of type I and type II interferon signaling on TNF-α production during H. pylori priming**

Monocytes were pre-treated without (w/o) or with **a)** IFN-α, IFN-β (10 ng/mL each)**,** the interferon-α/β receptor (IFNAR) antagonist Anifrolumab (20 ng/mL), **b)** IFN-γ (20 ng/mL) for 15 min or **c)** IFN-α, IFN-β (10 ng/mL each) and IFN-γ (20 ng/mL) for 15 min, then unprimed (“-”) or primed with viable *H. pylori (Hp),* heat-killed *H. pylori* (*Hp*^heat^), PFA-inactivated *H. pylori* (*Hp*^PFA^) (MOI 5) for 24 h. TNF-α secretion after priming was measured via ELISA. Data are presented as mean ± SD of **a)** 9; **b)** 8; **c)** 9 individual donors from at least two independent experiments. For statistical analysis, RM two-way ANOVA with **a)** Tukey’s or **b-c)** Šídák's post-hoc test was performed; *p*** ≤ 0.01.
